# Awareness and Perception of Dental Undergraduates in Belagavi District Towards the Newly Proposed National Exit Test for India

**DOI:** 10.7759/cureus.56356

**Published:** 2024-03-18

**Authors:** Varkey Nadakkavukaran Santhosh, David A Coutinho, Anil Ankola, Kavitha Ragu, Laxmi Kabra, Yuvarani Kandasamy Parimala, Siva Shankkari

**Affiliations:** 1 Public Health Dentistry, KLE Vishwanath Katti Institute of Dental Sciences, KLE Academy of Higher Education and Research, Belagavi, IND

**Keywords:** awareness, perception, national exit test, dental students, dental education

## Abstract

Background: The National Dental Commission Bill of 2023 introduced the National Exit Test (NExT) as a common final examination for all dental graduates in India, granting them the license to practice. This study evaluated dental undergraduates' awareness and perceptions of the newly proposed NExT.

Methods: A self-administered questionnaire in English comprising 23 close-ended questions was used to assess the awareness and perception of the students. A pilot study was conducted to determine the sample size, and 510 students were selected using a simple random sampling technique. The survey was administered to students ranging from 1st to 4th-year undergraduates and interns from two dental colleges in the Belagavi District, India. The questionnaire demonstrated good reliability (Cronbach's alpha coefficient = 0.86) and a content validity ratio 0.82.

Results: Interns had the highest mean awareness (39.56 ± 8.99) and perception (40.87 ± 5.56) scores, whereas first-year students had the lowest, with statistically significant differences among the groups (p ≤ 0.001). Although 81% of students were aware of NExT in India, only 17.3% found it student-friendly. A positive correlation was seen between the perception and awareness scores (r = + 0.242; p ≤ 0.001). The dependence of awareness and perception scores on predictors such as age, gender, and year of study were 16.7% and 15.3%, respectively.

Conclusion: Interns displayed a positive perception and higher awareness of NExT, whereas first and second-year dental students exhibited lower awareness and apprehensive perceptions. The introduction of NExT promises to enhance the overall quality of dental education on a national scale by providing high-quality care to patients.

## Introduction

Education imparts knowledge, skills, and values, while examinations assess comprehension and application, shaping modern learning by promoting critical thinking and problem-solving. Dental education in India has seen a shift from traditional theory-heavy exams to competency-based education that emphasizes practical skills, experiential learning, and patient-centered ethical approaches [1].

Dental education in India is regulated by the Dental Council of India (DCI), which ensures the maintenance of high dental education, training, and practice standards. However, like any evolving system, the Indian dental education landscape has challenges [2]. The increasing number of dental graduates and varying education standards across institutions have led to concerns about the quality and ethics of dental professionals entering the workforce [3]. The current mode of entry into postgraduation in dentistry is through the National Eligibility cum Entrance Test (NEET) conducted by the National Board of Examinations (NBE) [4]. NEET for Master of Dental Surgery (MDS) predominantly emphasizes the theoretical components of the curriculum, however a notable limitation of this approach is that it may not effectively evaluate the practical clinical skills of dental graduates [1]. Furthermore, dental practitioners aiming to establish private practices often encounter challenges in terms of credibility and recognition, as there is no standardized benchmark to measure dental professionals' ethical competency and expertise [5]. In this context, introducing the National Exit Test (NExT) emerges as a potential solution to address these concerns and reshape the trajectory of dental education and practice in India.

Recognizing the need to enhance oral healthcare quality and align dental education with global standards, the Indian parliament has passed the National Dental Commission (NDC) Bill, 2023 [6]. Among its key provisions, the bill introduces the concept of the NExT as a common final examination for all dental graduates to provide a license to practice [7]. This test aims to comprehensively evaluate dental graduates' knowledge, skills, and competencies before they are allowed to practice or pursue further education. The NExT is envisioned as a standardized examination that will replace the current entrance examination for postgraduates, ensuring a level playing field for all dental graduates. It will also serve as a benchmark to assess the proficiency of dental practitioners intending to set up private practices, thus enhancing the overall quality of oral healthcare delivery across the country [7].

In light of this context, this study seeks to shed light on dental undergraduates' awareness and perception of the recently introduced NExT in India. The primary objective of this investigation is to evaluate dental undergraduates' awareness and perception of the newly proposed NExT for India. By soliciting the perspectives of the individuals directly affected by the NExT, this study provides valuable insights capable of guiding policy decisions, facilitating curriculum development, and informing effective implementation strategies.

## Materials and methods

Study design and setting

This study employed a cross-sectional design and followed the STROBE reporting recommendations. This investigation was carried out on a sample of undergraduate students (ranging from 1st to 4th year) and interns from two dental colleges in Belagavi District, India.

Selection criteria

The inclusion criteria for this study included those students who were present during the day of data collection and those who provided informed consent. Those students who did not provide informed consent were excluded from the study.

Ethical clearance and informed consent

The study received ethical approval from the Institutional Research and Ethics Committee (SI No: 170, Dated: 05-09-2023). Official permission was obtained from the other dental colleges to conduct the study. All enrolled students were comprehensively briefed about the objectives of the study and voluntarily furnished written consent.

Questionnaire development

**Figure 1:**
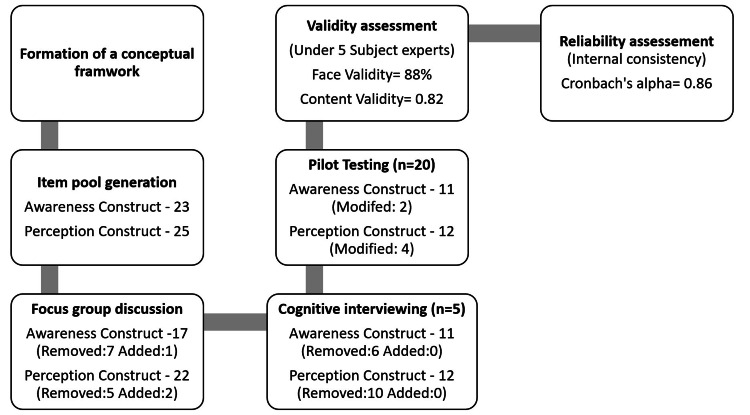
Steps involved in questionnaire development Awareness and perception constructs contain the initial pool of questions that developed after forming a conceptual framework.

The questionnaire was constructed using a six-stage method. A conceptual framework was formulated in the preliminary phase by synthesizing insights from literature and expert perspectives. This was followed by generating an item pool comprising 23 items designed to gauge the awareness construct and 25 items about the perception construct. In the subsequent phase, a focused group discussion involving eight subject matter experts was conducted, followed by cognitive interviews with a representative sample of five individuals. Across these stages, items were continually evaluated for contextual relevance, leading to selective inclusion and exclusion (Figure 1).

Pilot testing

A pilot investigation involved 20 undergraduate students (15 female, 5 male) to identify design inadequacies within the questionnaire. The students selected for the pilot study were representative of the target demographic. Specifically, two questions pertaining to awareness and four concerning perception were observed to lack clarity among the students. Subsequently, these identified questions were refined based on the feedback solicited from the students.

Validity and reliability assessments

Face validity was evaluated by calculating percentage agreement among a panel of five subject matter experts, yielding a substantial value of 88%. Furthermore, an appraisal of the content validity ratio was conducted for the questionnaire, revealing its efficacy as a valid instrument with a computed content validity ratio of 0.82. After this, the questionnaire's reliability was assessed employing Cronbach's alpha coefficient, yielding a robust estimate of 0.86, indicating a high level of internal consistency intrinsic to the questionnaire.

Sample size estimation and sample distribution

The sample size was determined based on findings from the pilot study, revealing that 40.3 % of female and 59% of male students exhibited low awareness of NExT, with a 3:1 allocation ratio for females to males. A minimum sample size of 492 students was obtained using G*Power software (Ver. 3.1.9.4.) with a Type-I (α) error of 0.05 and Power (1-β) of 0.95, and the final sample size was set at 510. An exhaustive list of undergraduate students from two dental colleges in Belagavi was utilized as the sampling frame, and they were selected through a simple random sampling technique employing the random number table method.

Questionnaire characteristics

The study employed a self-administered questionnaire consisting of 23 close-ended questions presented in English. The questionnaire encompassed two distinct sections: the first section gathered sociodemographic information from the students, while the second section delved into their awareness and perception of NExT. Among the 23 questions, 11 pertained to awareness, while the remaining 12 focused on perception. Responses were captured using a 5-point psychometric Likert scale, ranging from 1 to 5, and cumulative scores were computed for each question related to awareness and perception. Students with mean scores above 33 in awareness and above 36 in perception are considered to be both aware of and possess a positive perception of NExT (Appendix).

Data collection

A sole investigator administered the physical questionnaire within the operational hours of the two dental colleges. The questionnaire was disseminated in accordance with the respective academic years and conducted in a classroom environment on a designated date and time. Volunteers diligently monitored the process to safeguard against potential bias during classroom administration. Students were given explicit instructions to address all questions within a stipulated 15 minutes.

Statistical analysis

The collected data were entered into Microsoft Excel 2019 and analyzed using IBM-SPSS Statistics (Version 21, IBM Corp., Armonk, NY, USA). Descriptive statistics were computed, including percentages, means, and standard deviations. The Kolmogorov-Smirnov test confirmed the normal distribution of the data. The chi-square test assessed any significant association among the study variables. Unpaired student t-tests and Analysis Of Variance (ANOVA) tests were performed to identify significant differences among the study groups. Pearson correlation and multiple linear regression analysis were also performed. For all tests, a confidence level and level of significance were set at 95% and 5%, respectively.

## Results

The demographic profile of the dental students is presented in (Table 1). Among the 510 students, a significant proportion were females (61%). The mean age of the students was 21.75 ± 2.25.

**Table 1:** Demographic profile of the students SD: Standard Deviation; Values are expressed as frequency with percentages (in parentheses) and mean ± SD

Demographics variables	Frequency, % (N = 510)
Gender	
Male	199 (39%)
Female	311 (61%)
Year of Study	
1^st^ year	111 (21.8%)
2^nd^ year	101 (19.8%)
3^rd^ year	100 (19.6%)
4^th^ year	93 (18.2%)
Internship	105 (20.6%)
Age	Mean ± SD
1^st^ Year	19.84 ± 1.77
2^nd^ Year	20.32 ± 0.98
3^rd^ Year	21.65 ± 1.51
4^th^ Year	22.33 ± 1.73
Internship	24.73 ± 0.79
Total	21.75 ± 2.25

The frequency distribution of the responses from both awareness and perception-based questions are presented in (Tables 2-3). When the Chi-square test was employed, it was determined that all the questions exhibited statistically significant associations with the year of study. The majority (81%) of the students were aware of NExT, while the majority (43.7%) were unaware of the NDC bill. A smaller percentage (17.3%) of students expressed agreement on NExT being student-friendly. However, the majority (44.1%) of students felt that NExT would result in a significant shift in traditional teaching and studying methods.

**Table 2:** Frequency distribution of responses to awareness-based questions among the students All values are expressed as the frequency with percentages (in parentheses). The statistical test used: Chi-square test; Level of significance: *P ≤ 0.05 is considered a statistically significant association.

Question	Very unaware (%)	Unaware (%)	Neither aware or unaware (%)	Aware (%)	Very aware (%)	P-Value
Are you aware of India's upcoming National Exit Test (NExT)?	19 (3.7%)	30 (5.9%)	25 (4.9%)	413 (81.0%)	23 (4.5%)	0.001*
Are you aware about the purpose of NExT?	25 (4.9%)	112 (22.0%)	53 (10.4%)	30 (59.6%)	16 (3.1%)	0.001*
Are you aware that NExT will become mandatory for dental undergraduates before they begin their clinical practice in India?	36 (7.1%)	112 (22.0%)	43 (8.4%)	230 (45.1%)	89 (17.5%)	0.001*
Are you aware that NExT will replace the current National Eligibility cum Entrance Test (NEET) for Masters in Dental Surgery (MDS)?	43 (8.4%)	97 (19.0%)	57 (11.2%)	212 (41.6%)	101 (19.8%)	0.001*
Are you aware that NExT will act as a single official licentiate exam for clinical practice in India?	30 (5.9%)	116 (22.7%)	57 (11.2%)	290 (56.9%)	17 (3.3%)	0.001*
Are you aware that NExT will be conducted in two parts?	37 (7.3%)	287 (56.3%)	18 (3.5%)	163 (32.0%)	5 (1.0%)	0.001*
Are you aware that final-year undergraduates must qualify for NExT part I to be eligible for a rotatory internship?	60 (11.8%)	168 (32.9%)	34 (6.7%)	234 (45.9%)	14 (2.7%)	0.001*
Are you aware of the NExT examination pattern?	87 (17.1%)	316 (62.0%)	49 (9.6%)	58 (11.4%)	0 (0.0%)	0.001*
Are you aware that NExT will assess your clinical knowledge and practical hands-on skills to treat patients?	63 (12.4%)	195 (38.2%)	38 (7.5%)	205 (40.2%)	9 (1.8%)	0.001*
Are you aware that NExT parts one and two will be conducted twice a year?	77 (15.1%)	277 (54.3%)	40 (7.8%)	112 (22.0%)	4 (0.8%)	0.001*
Are you aware of the National Dental Commission Bill, 2023?	81 (15.9%)	223 (43.7%)	60 (11.8%)	61 (12.0%)	85 (16.7%)	0.001*

**Table 3:** Frequency distribution of responses to perception-based questions among the students All values are expressed as the frequency with percentages (in parentheses). The statistical test used: Chi-square test; Level of significance: *P ≤ 0.05 is considered a statistically significant association. NEET: National Eligibility cum Entrance Test; MDS: Master of Dental Surgery; NExT: National Exit Test

Question	Strongly Disagree (%)	Disagree (%)	Neither Agree or Disagree (%)	Agree (%)	Strongly agree (%)	P-Value
Do you feel NExT will be student-friendly?	126 (24.7%)	96 (18.8%)	179 (35.1%)	88 (17.3%)	21 (4.1%)	0.001*
Do you feel NExT will have an influence on students’ academic performance?	41 (8.0%)	59 (11.6%)	109 (21.4%)	190 (37.3%)	111 (21.8%)	0.001*
Do you feel NExT should be a compulsory requirement for all dental undergraduates?	63 (12.4%)	99 (19.4%)	229 (44.9%)	91 (17.8%)	28 (5.5%)	0.001*
Do you feel that NExT will be a better replacement for NEET MDS?	58 (11.4%)	118 (23.1%)	225 (44.1%)	101 (19.8%)	8 (1.6%)	0.001*
Do you feel NExT will decrease the number of dropouts in dental education?	45 (8.8%)	50 (9.8%)	105 (20.6%)	163 (32.0%)	147 (28.8%)	0.001*
Do you feel NExT will decrease stress and pressure to perform among dental students?	43 (8.4%)	79 (15.5%)	74 (14.5%)	162 (31.8%)	152 (29.8)	0.001*
Do you think NExT will enhance the overall quality of dental education?	46 (9.0%)	75 (14.7%)	202 (39.6%)	178 (34.9%)	9 (1.8%)	0.001*
Do you believe that NExT will ensure that there is an equal and standardized education system throughout the country?	126 (24.7%)	32 (6.3%)	163 (32.0%)	176 (34.5%)	13 (2.5%)	0.001*
Do you believe that NExT will filter those students who have successfully passed the exams but may lack the necessary hands-on skills to treat patients in clinical practice?	29 (5.7%)	37 (7.3%)	225 (44.1%)	193 (37.8%)	26 (5.1%)	0.001*
Do you believe that NExT will accurately assess student’s clinical knowledge and practical hands-on skills?	45 (8.8%)	55 (10.8%)	209 (41.0%)	185 (36.3%)	16 (3.1%)	0.001*
Do you think the idea of a single official licentiate exam such as NExT will be beneficial?	40 (7.8%)	97 (19.0%)	208 (40.8%)	148 (29.0%)	17 (3.3%)	0.001*
Do you feel that NExT will result in a significant shift in traditional teaching and studying methods?	15 (2.9%)	18 (3.5%)	149 (29.2%)	225 (44.1%)	103 (20.2%)	0.001*

The mean awareness and perception scores of students towards NExT are presented in (Table 4). The mean awareness scores were highest among interns (39.56 ± 8.99) and lowest among first years (29.54 ± 7.89), and it showed a statistically significant difference among the students (P ≤ 0.001) using the ANOVA test. Similarly, the mean perception scores were highest among interns (40.87 ± 5.56) and lowest among the first years (34.22 ± 8.03), which also showed a statistically significant difference among the students (P ≤ 0.001) using the ANOVA test.

**Table 4:** Distribution of mean perception and awareness scores based on gender and year of study among the students SD: Standard Deviation; All Values are expressed as mean ± SD; The statistical test used: α Unpaired student t-test and ψ Analysis of variance Test; Level of significance: *P ≤ 0.05 is considered statistically significant.

Study Characteristics	Awareness score (Mean ± SD)	P
Gender^α^		
Male	34.43 ± 8.87	0.008*
Female	31.96 ± 8.20	
Year of Study^ψ^		
1^st^ Year	29.54 ± 7.89	≤0.001*
2^nd^ Year	30.96 ± 8.16	
3^rd^ Year	30.18 ± 6.25	
4^th^ Year	34.53 ± 6.66	
Internship	39.56 ± 8.99	
Study Characteristics	Perception score (Mean ± SD)	P
Gender^α^		
Male	37.30 ± 6.80	0.993
Female	38.49 ± 6.87	
Year of Study^ψ^		
1^st^ Year	34.22 ± 8.03	≤0.001*
2^nd^ Year	36.10 ± 7.18	
3^rd^ Year	38.82 ± 4.52	
4^th^ Year	40.60 ± 5.68	
Internship	40.87 ± 5.56	

When Pearson's correlation coefficient test was applied, a positive linear correlation that was statistically significant was found between the awareness and perception scores (r = + 0.242; P ≤ 0.001). When multiple regression analysis was performed, it was found that the dependence of the awareness and perception scores on predictors such as age, gender, and the year of study were found to be 16.7% and 15.3%, respectively (Table 5).

**Table 5:** Association between demographic variables and perception/awareness scores of the students CI: confidence interval; SE: Standard error; The statistical analysis used: Multiple linear regression analysis; Level of significance: *P ≤ 0.05 is considered statistically significant.

Predictors	Coefficient r	SE	t	95% CI	P	Adjusted R^2^
Dependent Variable: Awareness score						
Constant	-	0.333	4.984	14.063-32.366	<0.001*	0.167
Age	0.082	0.106	1.326	-0.150-0.775	0.185	
Gender	-0.100	0.127	-2.445	-3.151- -0.343	0.015	
Year of Study	0.326	0.045	5.282	1.215-2.654	<0.001*	
Dependent Variable: Perception score						
Constant	-	3.773	9.610	28.841–43.665	<0.001*	0.153
Age	-0.11	0.191	-1.769	-0.712-0.037	0.078	
Gender	0.112	0.579	2.712	0.432-2.707	0.007	
Year of Study	0.467	0.297	7.494	1.640-2.805	<0.001*	

## Discussion

Dental education in India is progressing towards global standards; however, dental graduates encounter notable challenges, notably a deficiency in legislative updates. The dental curriculum has not changed since 2007 [8]. This has led to a crisis with limited job opportunities and insufficient recognition for specialized roles within dentistry, resulting in postgraduate unemployment [3,9]. In India, the proposed NExT under the NDC Act holds dual significance. Primarily, it is envisaged as a common exit and licentiate examination applicable to all Indian dental graduates. Secondly, it will be an evaluation tool for postgraduate selections across various dental specialties [10]. The present study aims to assess dental undergraduates' awareness levels and perceptions towards the newly proposed NExT for India.

Licentiate examinations are globally present, with prominent examples such as the United States Medical Licensing Examination and the National Medical Practitioners Qualifying Examination in Japan [11,12]. These examinations serve as essential gateways for assessing the competence of prospective healthcare practitioners, particularly their ability to provide ethical patient care [13]. NExT for dental education in India aims to achieve similar outcomes. In this study, a substantial majority (81%) of students demonstrated awareness of the impending introduction of NExT in India. Furthermore, a significant proportion exhibited knowledge of the overarching objectives associated with NExT. However, it is imperative to highlight that a notable proportion (35.1%) of the students expressed uncertainty regarding the extent to which NExT would be conducive to a student-friendly environment.

Furthermore, a majority (56.9%) of the students are acquainted with the idea of NExT as the country's exclusive official licentiate examination for clinical practice. In a study conducted by Bains et al., most students believe there is a compelling need to update the dental curriculum in alignment with current trends [2]. In this study, a substantial majority (44.1%) of students perceived that the implementation of NExT would bring about a significant transformation in traditional teaching and learning methodologies. These findings underscore the importance of considering student perspectives and adapting dental education to evolving educational paradigms.

Stress among dental students in India is a pressing issue, primarily stemming from concerns about the demanding daily schedule and fear of failing academic years, as identified in previous studies [14]. Furthermore, intricate clinical cases, uncooperative patients, and meeting clinical requirements add to the stress faced by dental students [15]. The anticipation surrounding the NExT initiative introduces another layer of complexity. Most students (61.6%) believed that NExT is poised to decrease stress levels and reduce the pressure to excel among dental students. Furthermore, a substantial majority (37.3%) of students perceived that NExT is likely to exert an influential impact on the academic performance of these students. These findings highlight that NExT is poised to bring down the stress among the participants as it will reduce the added pressure to excel in their exams. This sheds light on the significance of examining the potential ramifications of NExT on the stress levels and academic outcomes of dental students in India.

In rural India, there is approximately one dentist for every 250,000 people. This situation has led to a rise in quackery and unethical dental practice, particularly among lower-middle and lower socioeconomic classes who cannot afford qualified dental practitioners [16]. NExT will ensure only competent dental students begin their clinical practice throughout India. This study revealed a degree of uncertainty among students, as 41% are unsure about the capacity of NExT to accurately assess clinical knowledge and practical hands-on skills. A significant portion of the surveyed students (45.1%) were aware that NExT would be a prerequisite for dental undergraduates before commencing their clinical practice.

Similarly, 40.8% express reservations about the concept of a single official licentiate examination like NExT being beneficial. On a positive note, 40.2% of the students were aware of the primary objective of NExT, which was to evaluate their proficiency in clinical knowledge and practical skills that are ethical for patient care. These findings highlight skepticism among dental undergraduates regarding the impending role of NExT in shaping their clinical practice journey.

NEET-MDS is an eligibility-cum-ranking examination prescribed as the single entrance examination conducted by NBE for admission to various MDS Courses under the Dentists Act of 1948 [4,17]. Dentistry is a highly practical field where the ability to diagnose and treat patients effectively is crucial. NEET-MDS predominantly focuses on testing the theoretical knowledge of students through multiple-choice questions. It does not assess dental graduates' practical skills, clinical acumen, or hands-on experience [18,19]. A significant majority of surveyed students (41.6%) are aware of the forthcoming transition where the NExT will supplant the existing NEET MDS as the gateway to dental postgraduate programs.

Moreover, a larger majority (44.1%) were unsure that NExT represents a superior alternative to NEET MDS for this purpose. These findings contradict the results of a study conducted by Ingawale et al., wherein most medical students indicated that they believed there was no necessity to replace the current NEET PG examination [20]. However, a majority (44.9%) in this study expressed uncertainty about NExT being mandatory for all dental undergraduates. These findings emphasize that many students were uncertain whether NExT could effectively replace NEET MDS for postgraduate admissions.

NExT for dental is expected to follow its medical counterpart. It will be a two-part exam, with the first part being a theory exam and the second part being a practical exam. Students must clear both parts with a minimum passing score of 50%. Those who clear NExT will be eligible to register as dental practitioners in India and to apply for postgraduate dental courses [21]. In this study, a significant majority (62%) of the surveyed students lacked knowledge about the examination pattern employed by NExT.

Additionally, a substantial 56.3% were unaware that NExT would be divided into two distinct parts. Regarding eligibility, 45.9% of respondents knew that completing NExT Part I is a prerequisite for final-year undergraduates aiming to embark on their rotatory internship. Finally, 54.3% of students were unaware that both parts of NExT would be conducted semi-annually. These findings underscore the need for greater dissemination of information on NExT among dental undergraduates through proper channels, maybe through the state and central agencies of DCI.

Dropouts in dental education are a significant concern with multifaceted causes such as academic challenges and burnout [22]. Addressing this issue is crucial for aspiring dental professionals to reach their career goals and maintain a robust and skilled dental workforce in India. In this study, a notable proportion (39.6%) of students remain uncertain about whether NExT will contribute to an overall improvement in the quality of dental education. However, a significant majority (32%) expressed that it could lead to a decrease in the dropout rate within dental education in India. NExT for dental students offers a standardized evaluation mechanism, ensuring student competency and confidence, thereby reducing attrition rates and bolstering retention within dental education programs. Providing a clear proficiency benchmark incentivizes students to remain committed to their studies, minimizing the likelihood of dropout. Meanwhile, a substantial portion (34.5%) of students agreed that it would effectively establish an equal and standardized educational system nationwide.

An excellent opportunity lies within implementing the practical component of NExT part 2 to standardize the instruction of soft skills such as chairside manners, ethical principles, obtaining informed consent, addressing sensitive topics, and delivering difficult treatments [23]. There is a prevailing sense of uncertainty (44.1%) among students in this study regarding its ability to filter out individuals who may have passed exams but lack the essential practical skills required for clinical patient care. These diverse perspectives highlight the complexity surrounding NExT and its potential implications for dental education, encompassing concerns, uncertainties, and expectations for change.

The NDC Act of 2023 is a landmark piece of legislation that aims to overhaul India's dental education and professional landscape. It repealed the Dentists Act of 1948 and established the NDC as the new regulatory authority for dentistry. The NDC Act is expected to have a significant positive impact on dental education and practice in India [6]. The majority (43.7%) of the students in this study were unaware of the NDC bill.

The findings of this study have several implications for dental education in India. The evolving perceptions of students indicate that stakeholders should consider adopting a phased approach to implement NExT [24]. This study provides the knowledge essential for policymakers, educational institutions, and other stakeholders in the dental sector to make informed decisions and ensure that the transformation of dental education in India effectively addresses the pressing issues faced by dental graduates and aspiring professionals.

Limitations

This study was limited to two specific dental institutions in the country, which may not fully represent the entire population. A more comprehensive investigation involving a larger number of institutions nationwide could provide a broader perspective on NExT. Additionally, the questionnaire-based design might introduce response bias among students, which is an important consideration.

## Conclusions

This study highlights dental undergraduates' awareness and perception of the proposed NExT in India. The findings indicate that interns generally perceived NExT positively and showed greater awareness. In contrast, first- and second-year dental students exhibited significantly lower awareness and harbored apprehensive perceptions of NExT. It continues to shape the landscape of dental education in India. Policymakers and institutions should align their strategies with these findings to enhance training and benefit the healthcare system in India.

## Appendices

Awareness questions

1. Are you aware of the upcoming National Exit Test (NExT) in India?

a)  Very unaware       b) Unaware        c) Neither aware or unaware         d) Aware          e) Very aware

2.  Are you aware about the purpose of NExT?

a)  Very unaware       b) Unaware        c) Neither aware or unaware         d) Aware          e) Very aware

3. Are you aware that NExT will become mandatory for dental undergraduates before they begin their clinical practice in India?

a)  Very unaware       b) Unaware        c) Neither aware or unaware         d) Aware          e) Very aware

4. Are you aware that NExT will replace the current National Eligibility cum Entrance Test (NEET) for Masters in Dental Surgery (MDS)?

a)  Very unaware       b) Unaware        c) Neither aware or unaware         d) Aware          e) Very aware

5. Are you aware that NExT will act as single official licentiate exam for clinical practice in India?

a)  Very unaware       b) Unaware        c) Neither aware or unaware         d) Aware          e) Very aware

6. Are you aware that NExT will be conducted in two parts?

a)  Very unaware       b) Unaware        c) Neither aware or unaware         d) Aware          e) Very aware

7. Are you aware that final year undergraduates must qualify NExT part I to be eligible for rotatory internship?

a)  Very unaware       b) Unaware        c) Neither aware or unaware         d) Aware          e) Very aware

8. Are you aware of the examination pattern for NExT?    

a)  Very unaware       b) Unaware        c) Neither aware or unaware         d) Aware          e) Very aware

9. Are you aware that NExT will assess your clinical knowledge and practical hands-on skills to treat patients? 

a)  Very unaware       b) Unaware        c) Neither aware or unaware         d) Aware          e) Very aware

10. Are you aware that NExT part one and two will be conducted twice a year?    

a)  Very unaware       b) Unaware        c) Neither aware or unaware         d) Aware          e) Very aware

11. Are you aware of the National Dental Commission Bill, 2023?

a)  Very unaware       b) Unaware        c) Neither aware or unaware         d) Aware          e) Very aware

Perception questions

1. Do you feel NExT will be student friendly?

a) Strongly Disagree    b) Disagree    c) Neither agree or disagree      d) Agree          e) Strongly Agree

2. Do you feel NExT will have an influence on students’ academic performance?

a) Strongly Disagree    b) Disagree    c) Neither agree or disagree      d) Agree          e) Strongly Agree

3. Do you feel NExT should be a compulsory requirement for all dental undergraduates?

a) Strongly Disagree    b) Disagree    c) Neither agree or disagree      d) Agree          e) Strongly Agree

4. Do you feel that NExT will be a better replacement for NEET MDS? 

a) Strongly Disagree    b) Disagree    c) Neither agree or disagree      d) Agree          e) Strongly Agree

5. Do you feel NExT will decrease the number of drop outs in dental education?

a) Strongly Disagree    b) Disagree    c) Neither agree or disagree      d) Agree          e) Strongly Agree

6. Do you feel NExT will decrease stress and pressure to perform among dental students?

a) Strongly Disagree       b) Disagree       c) Neither agree or disagree         d) Agree        e) Strongly Agree

7. Do you think NExT will enhance the overall quality of dental education?

a) Strongly Disagree       b) Disagree       c) Neither agree or disagree         d) Agree        e) Strongly Agree

8. Do you believe that NExT will ensure that there is equal and standardized education system throughout the country?

a) Strongly Disagree       b) Disagree       c) Neither agree or disagree         d) Agree        e) Strongly Agree

9. Do you believe that NExT will filter those students who have successfully passed the exams but may lack the necessary hands-on skills to treat patients in clinical practice?

a) Strongly Disagree       b) Disagree       c) Neither agree or disagree         d) Agree        e) Strongly Agree

10. Do you believe that NExT will accurately assess student’s clinical knowledge and practical hands-on skills?

a) Strongly Disagree       b) Disagree       c) Neither agree or disagree         d) Agree        e) Strongly Agree

11. Do you think the idea of a single official licentiate exam such as NExT will be beneficial? 

a) Strongly Disagree       b) Disagree       c) Neither agree or disagree         d) Agree        e) Strongly Agree

12. Do you feel that NExT will result in a significant shift in traditional teaching and studying methods?

a) Strongly Disagree       b) Disagree       c) Neither agree or disagree         d) Agree        e) Strongly Agree

## References

[1] Kabra L, Santhosh VN, Sequeira RN, Ankola AV, Coutinho D (2023). Dental curriculum reform in India: Undergraduate students' awareness and perception on the newly proposed choice based credit system. J Oral Biol Craniofac Res.

[2] Bains R, Parikh V, Pandey P (2023). Undergraduate students' perception of the Indian dental curriculum: A focus-group based, multi-centric questionnaire survey. J Oral Biol Craniofac Res.

[3] Yadav S, Rawal G (2016). The current status of dental graduates in India. Pan Afr Med J.

[4] (2023). National board examination in medical sciences. https://natboard.edu.in/viewnbeexam?exam=neetmds.

[5] Kakkar M, Pandya P, Kawalekar A, Sohi M (2015). Evidence and existence of dental education system. Int J Sci Study.

[6] (2023). National dental commission bill, 2023 passed by the Parliament to elevate dental education and healthcare standards. https://pib.gov.in/PressReleaseIframePage.aspx?PRID=1946875#.

[7] (2023). Public notice for national dental commission bill. https://main.mohfw.gov.in/sites/default/files/Public%20Notice%20for%20NDC.pdf.

[8] Kumar S, Dagli RJ, Mathur A, Jain M, Prabu D, Kulkarni S (2009). Perceived sources of stress amongst Indian dental students. Eur J Dent Educ.

[9] Gandhi UV (2014). Current status and expected future direction of the prosthodontic speciality in India. JPS Global workshop Kyoto 2012. J Prosthodont Res.

[10] Dulloo P, Kanitkar M (2022). National exit test: The medical faculty perspective-A pilot study. Natl Med J India.

[11] Khalil S, Jose J, Welter M (2023). The importance of USMLE step 2 on the screening and selection of applicants for general surgery residency positions. Heliyon.

[12] Nagasaki K, Nishizaki Y, Nojima M (2021). Validation of the General Medicine in-training examination using the professional and linguistic assessments board examination among postgraduate residents in Japan. Int J Gen Med.

[13] Sarkar S, Ranjan P (2021). What are the options to implement an undergraduate medical exit examination in India?. Natl Med J India.

[14] Acharya S (2003). Factors affecting stress among Indian dental students. J Dent Educ.

[15] Harikiran AG, Srinagesh J, Nagesh KS, Sajudeen N (2012). Perceived sources of stress amongst final year dental under graduate students in a dental teaching institution at Bangalore, India: A cross sectional study. Indian J Dent Res.

[16] Oberoi SS, Oberoi A (2015). Growing quackery in dentistry: An indian perspective. Indian J Public Health.

[17] (2023). Dental council of India. https://dciindia.gov.in/Dentistact1948.aspx.

[18] Pardeshi G, Lakade R, Adhav P (2012). MCI and NEET-PG: Understanding the point of view of medical graduates. Natl Med J India.

[19] Ozair A, Singh AB (2020). Why are India’s best medical graduates not preferring ENT for postgraduate training through NEET-PG?. Indian J Otolaryngol Head Neck Surg.

[20] Ingawale S, Suvvari TK, Anand S, Garg R, Edara L (2022). Ensuring a smooth and hassle-free implementation of National Exit Test (NEXT) exam: Feedback from a pan-India online cross-sectional survey. Glob J Med Stud.

[21] (2023). NMC, national exit test regulations, 2023. Regulations.

[22] Singh P, Aulak DS, Mangat SS, Aulak MS (2016). Systematic review: Factors contributing to burnout in dentistry. Occup Med (Lond).

[23] Dhar P, Nundy S (2021). The NExT challenge - The national exit test. J Med Evid.

[24] Bhattacharya S, Kartikeyan S, Yadav YR (2021). Perceptions of MBBS students regarding the national exit test. Int J Physiol Nutr Phys Educ.

